# Integrating pulmonary and systemic transcriptomes to characterize lung injury after pediatric hematopoietic stem cell transplant

**DOI:** 10.1172/jci.insight.194440

**Published:** 2025-07-22

**Authors:** Emma M. Pearce, Erica Evans, Madeline Y. Mayday, Gustavo Reyes, Miriam R. Simon, Jacob Blum, Hanna Kim, Jessica Mu, Peter J. Shaw, Courtney M. Rowan, Jeffery J. Auletta, Paul L. Martin, Caitlin Hurley, Erin M. Kreml, Muna Qayed, Hisham Abdel-Azim, Amy K. Keating, Geoffrey D.E. Cuvelier, Janet R. Hume, James S. Killinger, Kamar Godder, Rabi Hanna, Christine N. Duncan, Troy C. Quigg, Paul Castillo, Nahal R. Lalefar, Julie C. Fitzgerald, Kris M. Mahadeo, Prakash Satwani, Theodore B. Moore, Benjamin Hanisch, Aly Abdel-Mageed, Dereck B. Davis, Michelle P. Hudspeth, Greg A. Yanik, Michael A. Pulsipher, Christopher C. Dvorak, Joseph L. DeRisi, Matt S. Zinter

**Affiliations:** 1 Division of Critical Care Medicine, Department of Pediatrics, University of California, San Francisco, San Francisco, California, USA.; 2 Departments of Laboratory Medicine and Pathology, Yale School of Medicine, New Haven, Connecticut, USA.; 3 The Children’s Hospital at Westmead, Westmead, New South Wales, Australia.; 4 Pediatric Transplantation and Cell Therapy Consortium.; 5 Indiana University, Department of Pediatrics, Division of Critical Care Medicine, Indianapolis, Indiana, USA.; 6 Hematology/Oncology/BMT and Infectious Diseases, Nationwide Children’s Hospital, Columbus, Ohio, USA.; 7 Center for International Blood and Marrow Transplant Research (CIBMTR), National Marrow Donor Program/Be The Match, Minneapolis, Minnesota, USA.; 8 Division of Pediatric and Cellular Therapy, Duke University Medical Center, Durham, North Carolina, USA.; 9 Division of Critical Care, Department of Pediatric Medicine, St. Jude Children’s Research Hospital, Memphis, Tennessee, USA.; 10 Department of Child Health, Division of Critical Care Medicine, University of Arizona, Phoenix, Arizona, USA.; 11 Aflac Cancer & Blood Disorders Center, Children’s Healthcare of Atlanta and Emory University, Atlanta, Georgia, USA.; 12 Department of Pediatrics, Division of Hematology/Oncology and Transplant and Cell Therapy, Keck School of Medicine, University of Southern California, Los Angeles, California, USA.; 13 Loma Linda University School of Medicine, Cancer Center, Children Hospital and Medical Center, Loma Linda, California, USA.; 14 Harvard Medical School, Division of Pediatric Oncology, Department of Pediatrics, Dana-Farber Cancer Institute and Boston Children’s Hospital, Boston, Massachusetts, USA.; 15 Center for Cancer and Blood Disorders, Children’s Hospital Colorado and University of Colorado, Aurora, Colorado, USA.; 16 CancerCare Manitoba, Manitoba Blood and Marrow Transplant Program, University of Manitoba, Winnipeg, Manitoba, Canada.; 17 University of Minnesota, Department of Pediatrics, Division of Critical Care Medicine, Minneapolis, Minnesota, USA.; 18 Division of Pediatric Critical Care, Department of Pediatrics, Weill Cornell Medicine, New York, New York, USA.; 19 Cancer and Blood Disorders Center, Nicklaus Children’s Hospital, Miami, Florida, USA.; 20 Department of Pediatric Hematology, Oncology and Blood and Marrow Transplantation, Pediatric Institute, Cleveland Clinic, Cleveland, Ohio, USA.; 21 Pediatric Blood and Marrow Transplantation Program, Texas Transplant Institute, Methodist Children’s Hospital, San Antonio, Texas, USA.; 22 Section of Pediatric BMT and Cellular Therapy, Helen DeVos Children’s Hospital, Grand Rapids, Michigan, USA.; 23 University of Florida, Gainesville, UF Health Shands Children’s Hospital, Gainesville, Florida, USA.; 24 Division of Pediatric Hematology/Oncology, UCSF Benioff Children’s Hospital Oakland, University of California San Francisco, Oakland, California, USA.; 25 Department of Anesthesiology and Critical Care, Perelman School of Medicine, Children’s Hospital of Philadelphia, University of Pennsylvania, Philadelphia, Pennsylvania, USA.; 26 Department of Pediatrics, Division of Hematology/Oncology, MD Anderson Cancer Center, Houston, Texas, USA.; 27 Division of Pediatric Hematology, Oncology and Stem Cell Transplantation, Department of Pediatrics, Columbia University, New York, New York, USA.; 28 Department of Pediatric Hematology-Oncology, Mattel Children’s Hospital, University of California, Los Angeles, California, USA.; 29 Children’s National Hospital, Washington DC, USA.; 30 Department of Pediatrics, Hematology/Oncology, University of Mississippi Medical Center, Jackson, Mississippi, USA.; 31 Adult and Pediatric Blood & Marrow Transplantation, Pediatric Hematology/Oncology, Medical University of South Carolina Children’s Hospital/Hollings Cancer Center, Charleston, South Carolina, USA.; 32 Pediatric Blood and Bone Marrow Transplantation, Michigan Medicine, University of Michigan, Ann Arbor, Michigan, USA.; 33 Division of Pediatric Hematology and Oncology, Intermountain Primary Children’s Hospital, Huntsman Cancer Institute, Spencer Fox Eccles School of Medicine at the University of Utah, Salt Lake City, Utah, USA.; 34 Division of Allergy, Immunology, and Bone Marrow Transplantation, Department of Pediatrics, and; 35 Department of Biochemistry and Biophysics, UCSF, San Francisco, California, USA.; 36 Chan Zuckerberg Biohub, San Francisco, California, USA.

**Keywords:** Immunology, Inflammation, Pulmonology, Bone marrow transplantation, Stem cell transplantation, Transcriptomics

## Abstract

We previously showed that bronchoalveolar lavage (BAL) transcriptomes representing pulmonary inflammation and cellular injury can phenotype post-HCT lung injury and predict mortality. To test whether peripheral blood might be a suitable surrogate for BAL, we compared 210 paired BAL and blood transcriptomes obtained from 166 pediatric patients with HCT at 27 hospitals. BAL and blood RNA abundance showed minimal correlation at the level of individual genes, gene set enrichment scores, imputed cell fractions, and T and B cell receptor clonotypes. Instead, we identified significant site-specific transcriptional programs. In BAL, pathways related to immunity, hypoxia, and epithelial mesenchymal transition were tightly coexpressed and linked to mortality. In contrast, in blood, expression of endothelial injury, DNA repair, and cellular metabolism pathways was associated with mortality. Integration of paired BAL and blood transcriptomes dichotomized patients into 2 groups with significantly different rates of hypoxia and clinical outcomes within 1 week of BAL. These findings reveal a compartmentalized injury response, where BAL and blood transcriptomes provide distinct but complementary insights into local and systemic mechanisms of post-HCT lung injury.

## Abstract 2

In stem cell transplant patients, paired lung and peripheral blood transcriptomes show little correlation, instead revealing a compartmentalized response with distinct yet complementary gene expression signatures of post-transplant lung injury.

## Introduction

Hematopoietic stem cell transplantation (HCT) combines high-dose chemotherapy and/or radiation with i.v. infusion of hematopoietic progenitor cells in order to eradicate and replace malignant or dysfunctional cellular lineages (1). While the number of HCT procedures has increased dramatically and outcomes have improved over time, safety remains a concern. Specifically, acute or chronic lung injury can develop in 20%–40% of patients due to chemotherapy toxicity, infections, and/or immune dysregulation (2, 3). Post-HCT lung injury can lead to impaired quality of life and premature death (4, 5). Treatments largely include supportive care, antiinfectives, and attempts at immune modulation where possible but are limited by a need for deeper understanding of the lung microenvironment after HCT (6).

Therefore, the Pediatric Transplant and Cell Therapy Consortium (PTCTC) undertook a broad cross-sectional study of patients undergoing bronchoalveolar lavage (BAL) as diagnostic evaluation for post-HCT lung injury. We measured pulmonary microbiomes and paired human lung gene expression in BAL samples and identified 4 lung injury subtypes with varying degrees of dysbiosis, infection, inflammation, and cellular injury (7). This approach challenged previous clinical diagnoses such as idiopathic pneumonia syndrome (IPS) by increasing detection of pathogens and clarifying dominant biologic processes in the lung. Importantly, clinical outcomes varied significantly across the 4 subtypes, providing a biology-guided framework for risk stratification in this population. For example, patients with depleted pulmonary microbiomes, upregulated T cell signaling, diminished alveolar macrophage activity, and signs of epithelial mesenchymal transition (EMT) had 3- to 4-fold higher in-hospital mortality than their counterparts.

It remains crucial to determine whether these processes are compartmentalized to the lung or are indicative of systemic disease, as this could affect the development of both precision medicine diagnostics and treatments. From a diagnostic standpoint, BAL is a medically invasive procedure that is limited to those with acceptably low illness severity and is not well suited for serial sampling to monitor disease evolution or treatment-response (8, 9). Thus, surrogates of lung biology obtained through noninvasive testing such as blood sampling could enable more nimble diagnosis and disease tracking. From a therapeutic standpoint, understanding whether and how these lung processes are reflected in or regulated by the blood compartment could inform whether eventual treatments ought to be delivered directly to the lung, through the blood, or both.

Thus, we undertook a systems biology approach to compare paired BAL and peripheral blood samples from pediatric patients with post-HCT lung injury. We questioned whether peripheral blood transcriptomes would correlate with key aspects of BAL transcriptomes that we had previously linked to disease evolution and clinical outcomes. Here we present an integrated approach to understanding BAL, blood, and combined signatures of post-HCT lung injury that advance our understanding of local and systemic disease processes after pediatric HCT.

## Results

### Patient characteristics

**Figure 1:**
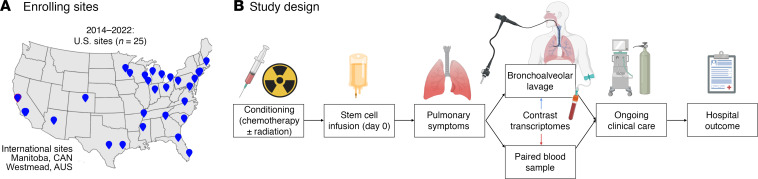
Study design. (**A**) Geographic location of participating children’s hospitals. (**B**) Patients were followed from the time of conditioning chemotherapy for the development of pulmonary symptoms. If bronchoscopy with bronchoalveolar lavage was planned for clinical reasons, patients were enrolled and BAL with a paired blood sample was collected. Patients were then followed clinically through hospital discharge.

As previously described, the PTCTC SUP1601 study enrolled 229 pediatric patients with HCT who developed post-HCT lung injury and underwent 278 clinically indicated BAL procedures (7). Patients were enrolled across 32 children’s hospitals in the United States, Canada, and Australia between 2014 and 2022. For this study, paired peripheral blood was collected during BALs and 210 BAL-blood pairs in 166 patients from 27 hospitals were included for analysis (Figure 1). Patients varied broadly in age, sex, race, ethnicity and geography; transplant indication was most commonly hematologic malignancy followed by receipt of mostly bone marrow or peripheral blood allografts from a variety of donor types (Table 1). Post-HCT lung injury signs and symptoms included hypoxia, dyspnea, declining pulmonary function testing, and/or abnormal chest imaging and often developed in conjunction with other complications such as graft versus host disease (GVHD; Table 2). BAL was performed a median 131 days after HCT (IQR, 37–376), at which point the cohort displayed a median absolute neutrophil count (ANC) of 3,042 cells/μL (IQR, 1,620–5,511) and a median absolute lymphocyte count (ALC) of 482 cells/μL (IQR, 184–1,159); approximately half of the cohort required supplemental oxygen at the time of sample collection. Based on BAL clinical microbiology results, lung injury was classified as lower respiratory tract infection (95 of 210), nonpulmonary sepsis (4 of 210), or IPS (111 of 210). After each patient’s most recent BAL, 85 of 166 patients required intensive care (51%), 48 required ≥ 7 days of mechanical ventilation (29%), and 32 patients died prior to hospital discharge (19%).

### Contrasting transcriptomes in paired BAL and peripheral blood

**Figure 2:**
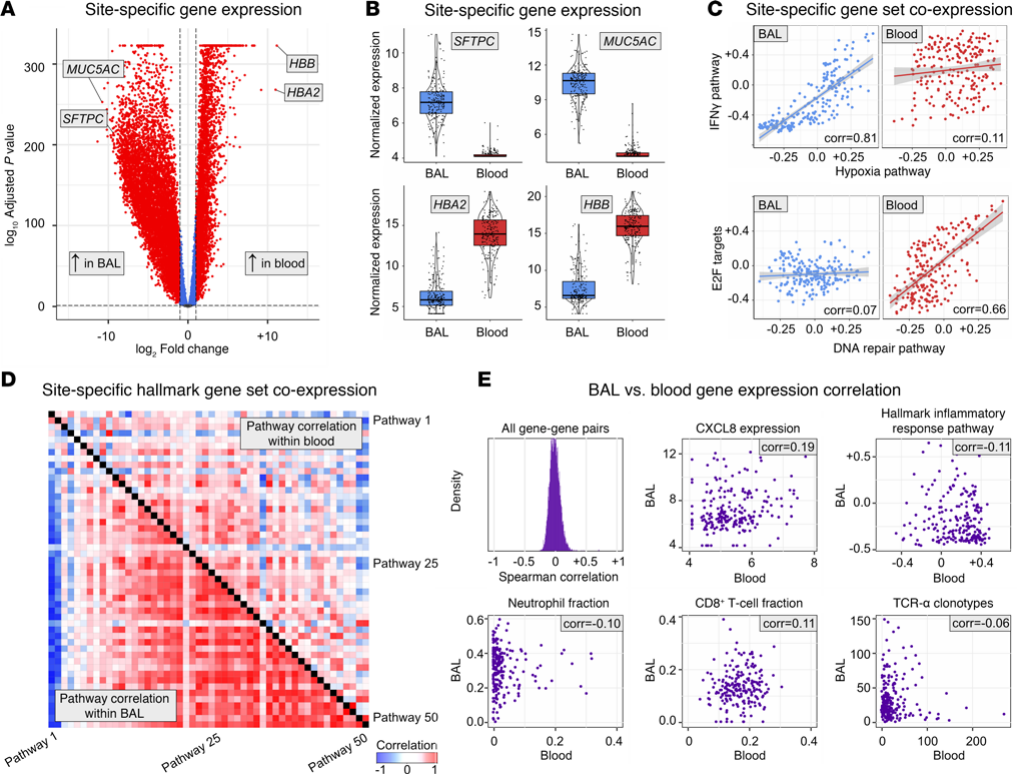
Differential gene expression and coexpression in BAL and paired blood samples. (**A**) Genes differentially expressed in BAL versus peripheral blood are shown. (**B**) Expression levels of select genes specific to lung (SFTPC, MUC5AC) and blood (HBA2, HBB). (**C**) Gene set enrichment scores to the 50 MSigDB Hallmark Pathways were calculated, and correlation between expression of each gene set was calculated within each body site and then contrasted to identify site-specific coregulation. Here we show unique coexpression of Hallmark Hypoxia and IFN-γ gene sets in BAL but not blood as well as unique coexpression of Hallmark DNA Repair and E2F targets in blood but not BAL. (**D**) Correlation of MSigDB Hallmark pathways within blood (top right triangle) and within BAL (bottom left triangle) are shown. See Supplemental Figure 1 for detailed labels. (**E**) BAL-blood correlation was calculated for expression levels of *n* = 7,169 protein-coding genes, and the distribution of correlation coefficients is plotted. Expression levels of CXCL8 in BAL and blood are shown as an example of minimal correlation. Gene set enrichment scores for the MSigDB Hallmark Inflammatory Response gene set measured in BAL and blood are also shown as an example of minimal BAL-blood correlation. BAL and peripheral blood cell type fractions were imputed using CIBERSORTx and reference atlases, and cell fractions across body sites were correlated using Spearman correlation coefficients, with neutrophils and CD8^+^ T cells shown as examples. T and B cell receptor clonotypes were measured using ImReP, and the number of unique clonotypes across body sites were correlated using Spearman correlation coefficients, with TRA shown as an example.

We hypothesized that both BAL and peripheral blood could yield complementary insight into pulmonary disease states in this cohort. To contrast the information captured in each sample type, we first compared BAL and blood sample pairs. We observed tissue-specific differential gene expression, with 6,156 genes showing greater expression in BAL and 9,368 genes showing greater expression in blood (Figure 2A and Supplemental Data File 1; supplemental material available online with this article; https://doi.org/10.1172/jci.insight.194440DS1). For example, as expected, Surfactant Protein C (*SFTPC*) was expressed to a greater level in BAL, whereas Hemoglobin subunit Alpha 2 (*HBA2*) was expressed to a greater level in blood (Figure 2B). Many canonical lung-specific genes were detected in the blood of many patients (e.g., *SFTPC* 53/210, *MUC5AC* 92/210, *LAMP3* 180/210), albeit rarely above 1 read per million per sample.

To understand the different regulatory networks unique to each body site, we next tested for tissue-specific differential gene coexpression. When assessing enrichment scores to the 50 MSigDB Hallmark gene sets (10), differential coexpression networks revealed highly site-specific interactions (Supplemental Data File 2). Of note, 39 gene sets were coexpressed in BAL but not blood; for example, Hallmark Hypoxia expression scores were correlated with Hallmark IFN-α and IFN-γ signaling, DNA repair signaling, and oxidative phosphorylation signaling in the lung (correlation ≥ 0.5) but not in the blood (correlation < 0.1, FDR-adjusted *P* < 0.05). In contrast, 9 gene sets were coexpressed in blood but not BAL; for example, Hallmark DNA Repair expression scores were correlated with early region 2 binding factor (E2F) signaling in the blood (correlation ≥ 0.5) but not in the lung (correlation < 0.1, FDR-adjusted *P* < 0.05; Figure 2C). Thus, overall, BAL gene networks showed much stronger coexpression than blood gene networks (Figure 2D and Supplemental Figure 1), suggesting synchronization of biological pathways in the lung.

Given the site-specific differences in expression levels and coexpression networks, we next sought to determine the degree of cross–body site transcriptome correlation. For any given gene, there was minimal correlation between BAL and blood expression levels (median Spearman rho = –0.005; IQR, –0.056 to –0.002; Supplemental Data File 3 and Figure 2E). As an example, the lack of BAL-blood correlation for *CXCL8* gene expression is depicted in Figure 2E. Similar results were found when analyzing enrichment scores to broader gene networks in the MSigDB collection (Supplemental Data File 4 and Figure 2E) and when analyzing imputed cell fractions (Supplemental Data File 5 and Figure 2E). For example, the imputed fraction of neutrophils in BAL and blood sample pairs showed no correlation (Spearman correlation = –0.099, FDR-adjusted *P* = 0.520). Patient-level cell fractions in BAL and blood are depicted in Supplemental Figure 2 to demonstrate the lack of cross-site correlation. Of note, the blood ANC correlated with imputed blood neutrophil fraction (Spearman rho = 0.462, *P* < 0.001) but not BAL neutrophil fraction (Spearman rho=0.043, *P* = 0.549). We then calculated T and B cell receptor clonotypes using ImReP; we detected a median 16 TRA clonotypes in BAL (IQR, 8–31; range, 0–271) and 25 TRA clonotypes in blood (IQR, 13–42; range, 0–148) but no T-cell receptors alpha (TRA) clonotypes were shared across BAL-blood pairs (except 1 clonotype in 1 of 210 sample pairs). Similar results were found for T-cell receptors beta, gamma, and delta; immunoglobin heavy chain (IGH); kappa light chain (IGK); and lambda light chain (Supplemental Data File 6). Together, these data highlight the uniqueness of the biological information contained in both BAL and blood.

### BAL and peripheral blood transcriptomes differ by survival outcome

**Figure 3:**
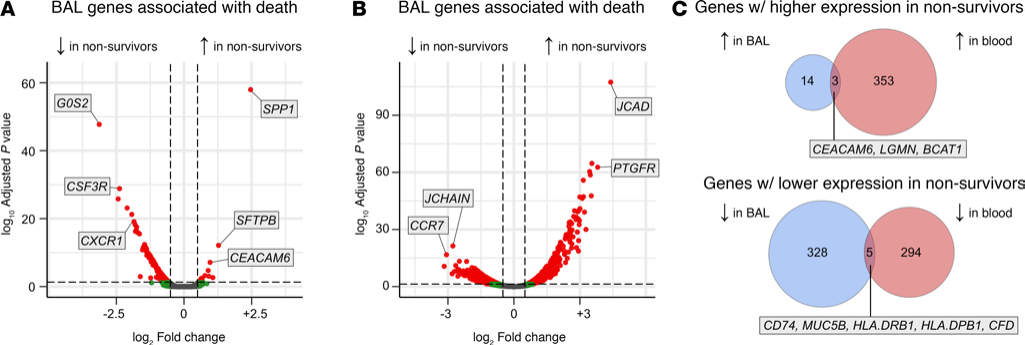
Differential gene expression by survival status. (**A**) BAL gene expression differences in nonsurvivors. (**B**) Peripheral blood gene expression differences in nonsurvivors. (**C**) Overlap between BAL and blood genes associated with mortality.

To better understand site-specific and cross-site contributors to survival, we next contrasted transcriptomes in survivors and nonsurvivors. We again observed tissue-specific differential gene expression in nonsurvivors. In BAL, expression levels of 350 genes were associated with death; overall, nonsurvivors had increased expression of genes related to epithelial injury and hypoxia (e.g., *SPP1*, *SFTPB*, *CEACAM6*) and lower expression of genes related to innate immune signaling (e.g., *CSF3R*, *CXCR1*, *IL1B*, *CXCL8*; Supplemental Data File 7 and Figure 3A). In blood, expression levels of 656 genes were associated with death; nonsurvivors had increased expression of genes related to endothelial cell junction and smooth muscle activity (e.g., *JCAD*, *PTGFR*) and lower expression of genes related to lymphocyte signaling (e.g., *JCHAIN*, *CCR7*; Supplemental Data File 8 and Figure 3B). Genes associated with death in BAL and in blood showed minimal overlap (Figure 3C), again indicating different site-specific contributors to outcome. Notably, *CEACAM6*, *LGMN*, and *BCAT1* showed increased expression in both BAL and blood of nonsurvivors, while *CD74*, *MUC5B*, *HLA*.*DRB1* and *HLA.DPB1*, and *CFD* showed lower expression in both BAL and blood of nonsurvivors.

**Figure 4:**
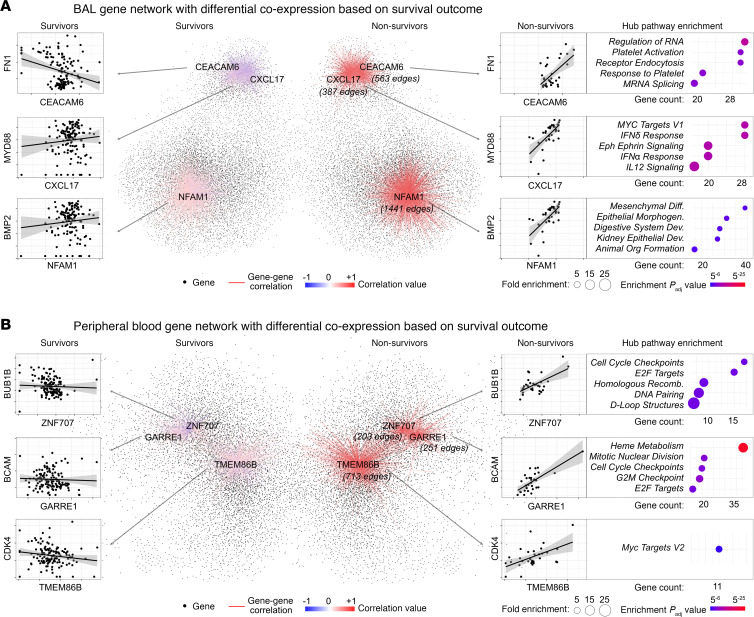
Differential gene coexpression by survival status. (**A**) Network of genes coexpressed in BAL of nonsurvivors (right) but not coexpressed in survivors (left). Examples of hubs genes (CEACAM6, CXCL17, NFAM1) are shown. Examples of differentially coexpressed genes linked to each hub gene are shown (e.g., CEACAM6-FN1 coexpression) to illustrate differential gene-expression. To the right, pathway enrichment for hub genes are shown. (**B**) Network of genes coexpressed in peripheral blood of nonsurvivors (right) but not coexpressed in survivors (left). Examples of hub genes (ZNF707, GARRE1, TMEM86B) are shown. Examples of differentially coexpressed genes linked to each hub gene are shown (e.g., ZNF707-BUB1B coexpression) to illustrate differential gene-expression. To the right, pathway enrichment for hub genes are shown.

In many diseases, genes maintain stable expression levels but exhibit altered coexpression patterns with other genes, suggesting that they influence pathobiology through network reorganization rather than heightened expression (11, 12). Therefore, we next tested for tissue-specific differential gene coexpression unique to nonsurvivors. In BAL, a network of 10,038 genes was coexpressed in nonsurvivors, but these genes were not coexpressed in survivors (Spearman rho ≥ 0.5 in nonsurvivors versus rho < 0.1 in survivors, FDR < 0.05; Supplemental Data File 9). Top hub genes included *CEACAM6*, which was coexpressed with 563 other genes related to platelet signaling and endoplasmic reticulum stress; *CXCL17*, which was coexpressed with 387 other genes related to immune signaling and hypoxia; and *NFAM1*, which was coexpressed with 1,441 genes related to EMT and lung morphogenesis (Figure 4A). In contrast, in blood, a network of 6,929 genes was correlated in blood of nonsurvivors but not survivors (Supplemental Data File 10). Top hub genes included *ZNF707*, which was coexpressed with 203 other genes representing cell cycle transitions and DNA repair, and *GARRE1*, which was coexpressed with 251 other genes representing heme metabolism (Figure 4B). These gene coregulatory networks complement the gene expression pathways noted above and accentuate the differences in biologic activity identified in BAL versus peripheral blood specimens.

Although we observed minimal BAL-blood transcriptome correlation in the overall cohort, we hypothesized that nonsurvivors might show greater cross-site correlation due to systemic illness and increased signaling across the alveolar-capillary membrane. However, we did not identify any gene sets that were differentially correlated between BAL and blood according to survivor status (all FDR-adjusted *P* > 0.1; Supplemental Data File 11). For example, Hallmark Hypoxia gene expression signaling in BAL and blood were minimally correlated in both survivors and nonsurvivors (*P* > 0.05), and these correlations were not significantly different (FDR-adjusted *P* = 0.994). This indicates that even the sickest patients do not show significant cross-site transcriptome correlation.

### BAL and peripheral blood transcriptomes differ by lung injury subtype

**Figure 5:**
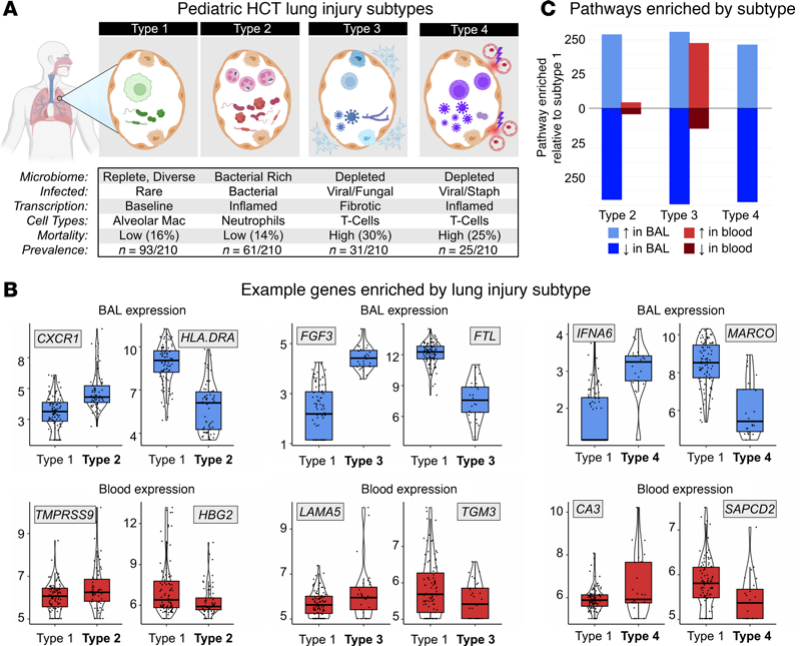
BAL and peripheral blood transcriptome correlates of post-HCT lung injury subtypes. (**A**) Concept diagram for 4 post-HCT lung injury subtypes derived in the PTCTC SUP1601 cohort and validated in the University of Utrecht, Netherlands, cohort (58). (**B**) Example BAL and blood genes differentially expressed in lung injury subtypes 2, 3, and 4 relative to subtype 1. (**C**) Differentially expressed BAL and blood genes underwent pathway analysis, and pathways identified in BAL and blood gene are quantified to show greater overall differences detected in BAL as opposed to paired blood.

We previously identified 4 subtypes of HCT-related lung injury based on BAL transcriptome-microbiome signatures (7, 13, 14). These subtypes varied not only in clinical outcomes but also in burden of infection, microbiome dysbiosis, inflammation, and cell injury (Figure 5A). Of note, lung injury Subtype 1 was defined by alveolar macrophage (AM) predominance, a replete and balanced microbiome, minimal signs of inflammation, and superior clinical outcomes. To better understand the biology of these lung injury subtypes, we next contrasted BAL and blood transcriptome pairs in reference to the best-performing Subtype 1, with results summarized in Figure 4 and Table 3 and described in detail below.

#### Subtype 2.

We previously showed that patients with Subtype 2 had higher rate of pulmonary bacterial infections and low in-hospital mortality. In BAL, Subtype 2 patients differentially expressed 2,027 genes (|LFC| ≥ 1, FDR-adjusted *P* < 0.05), including higher expression of genes related to granulocyte and inflammasome pathways (e.g., *CXCR1*, *IL1R2*, *S100A8*) and lower expression of genes related to macrophage and lymphocyte signaling (e.g., *CCL18*, *APOE*, *HLA.DRA*; Figure 5B). Supporting this, cell deconvolution showed an increase in BAL neutrophil fraction and a decrease in the AM fraction relative to Subtype 1 (Supplemental Data File 12). Patients with Subtype 2 had unique BAL coexpression of a network of 9,181 genes including top hubs *NLRP3*, *CXCR1*, and *ALPL*, which together showed enrichment for immune activation, cell-cell adhesion, platelet activity, and extracellular matrix interactions (Supplemental Data File 13). In peripheral blood, Subtype 2 patients differentially expressed 1,061 genes, including higher expression of genes related to thyroid activity (e.g., *CALCA*, *TPO*, *DIO2*) and lower expression of genes related to heme metabolism (e.g., *HBD*, *HBG2*) with no differences in blood cell fractions noted (Supplemental Data File 14). These patients also had greater blood coexpression of a network of 8,347 genes, including top hubs *NUP210*, *CDH13*, and *COL6A6*, which together represent DNA replication, oxidative phosphorylation, and cellular metabolism (Supplemental Data File 15). Comparison of enriched pathways in BAL and blood showed only 1 overlapping gene set: *Hallmark Heme Signaling* was lower in both BAL and blood of patients with Subtype 2. These data illustrate that, in patients with Subtype 2, pulmonary bacterial infections and a pulmonary neutrophil response are associated with systemic signs of increased blood cell metabolic activity without clear shifting of the fractions of blood cell types.

#### Subtype 3.

In contrast, patients with Subtype 3 showed a high rate of pulmonary microbiome depletion as well as high mortality. In BAL, Subtype 3 patients differentially expressed 8,166 genes, including higher expression of genes related to fibroblast activation, myogenesis, and nitric oxide signaling (e.g., *FGF3*, *BMP1*, *NOS*) and lower expression of genes related to macrophage signaling (e.g., *CD74*, *FTL*, *HLA*.*DRA*) and response to hypoxia (e.g., *HIF1A*, *HMOX1*; Figure 5C). In conjunction, cell deconvolution showed an increase in BAL CD4^+^ and CD8^+^ lymphocyte fractions and a decrease in the AM fraction relative to Subtype 1 (Supplemental Data File 16). Patients with Subtype 3 had unique BAL coexpression of a network of 6,724 genes, including top hubs *SIGLEC1*, *PTPRC*, and *HADHB*, which together showed enrichment for collagen deposition, integrin interactions, and EMT (Supplemental Data File 17). In peripheral blood, Subtype 3 patients differentially expressed 574 genes, including higher expression of genes related to collagen deposition and EMT (e.g. *COL4A1*, *LAMA5*, *TIMP3*), with no differences in blood cell fractions noted (Supplemental Data File 18). These patients also had greater blood coexpression of 9,269 genes, including top hubs *ANO6*, *GPD2*, and *TLR8*, which together represented DNA repair, T cell signaling, and heme metabolism (Supplemental Data File 19). Unique to Subtype 3, comparison of enriched pathways in BAL and blood showed substantial overlap for genes related to EMT and endothelial activation. These data illustrate that, in patients with Subtype 3, pulmonary microbiome depletion, T cell activation, and profibrotic signaling coocur with systemic signs of EMT.

#### Subtype 4.

Finally, patients with Subtype 4 showed both commensal microbiome depletion and viral infection, again with high mortality rates. In BAL, patients with Subtype 4 differentially expressed 6,252 genes, including higher expression of genes related to NK/T cell activity (e.g. *IL2*, *KLRF1*, *IFNG*, *IFNA6*; Figure 5B), β-defensins (*DEFB114*, *DEFB110*), and EMT (*COL11A1*, *MMP27*), and lower expression of genes related to AM signaling (e.g., *MARCO*, *FTH1*, *HLA*.*C*), neutrophil signaling (e.g., *MYD88*, *TREM2*), and airway and alveolar epithelial function (e.g., *SPRR3*, *MUC5B*, *SFTPB*). In conjunction, cell deconvolution showed an increase in BAL CD4^+^ and CD8^+^ lymphocyte fractions and a decrease in the AM fraction relative to Subtype 1 (Supplemental Data File 20). Patients with Subtype 4 had unique BAL coexpression of a network of 10,223 genes including top hubs *SLC38A6*, *ETFDH*, and *TAF2*, which together showed enrichment for collagen deposition, ankyrin interactions, and EMT (Supplemental Data File 21). In peripheral blood, patients with Subtype 4 differentially expressed only 156 genes, including weak overlap with IFN-α/β pathways (e.g., *OAS3*, *IFIT1*) and modest increase in blood CD4^+^ lymphocyte fraction (Supplemental Data File 22). These patients also had greater blood coexpression of 6,908 genes including top hubs *PRR16*, *SCML2*, and *TRPC6*, which together represented DNA repair, oxidative phosphorylation, and aerobic respiration (Supplemental Data File 23). Comparison of enriched pathways in BAL and blood showed no overlapping gene sets. These data illustrate that, in patients with Subtype 4, pulmonary microbiome depletion, T cell activation, and epithelial injury response coocur with nonspecific systemic signs of increased metabolic activity, but BAL and blood transcriptomes overall showed minimal overlap.

### Integrated BAL and blood transcriptome signatures

**Figure 6:**
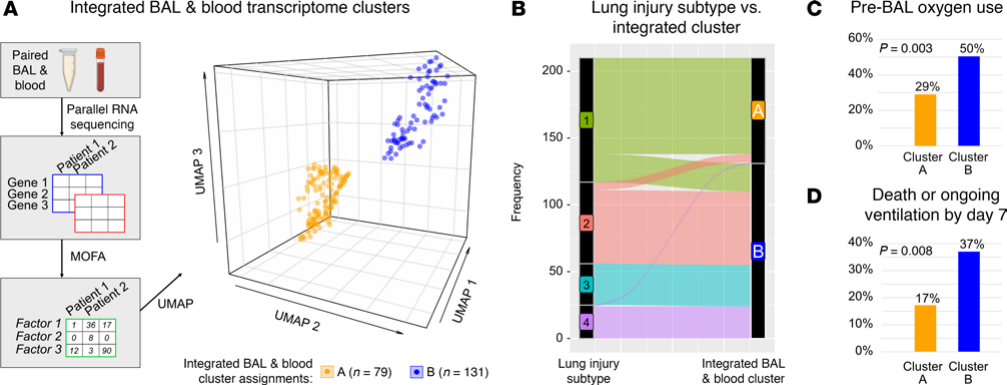
Integrated BAL and blood transcriptomic signatures reveal 2 large patient groups. (**A**) Paired BAL and blood transcriptomes underwent multi-omics factor analysis (MOFA) followed by dimensionality reduction (UMAP) and k-means clustering to show 2 groups of patients. (**B**) Post-HCT lung injury subtype was mapped onto the 2 integrated transcriptome clusters, showing that most patients from subtypes 2, 3, and 4 mapped to Cluster B. (**C** and **D**) Approximately twice as many patients in Cluster B required oxygen prior to BAL sampling, and twice as many patients died or required ongoing mechanical ventilation within 7 days of BAL sampling.

Given that paired BAL and blood transcriptomes were not correlated, we next questioned whether they would complement each other in understanding patient illness and clinical outcomes. By inputting normalized BAL and blood transcriptomes into multi-omics factor analysis (MOFA) followed by dimensionality reduction (Uniform Manifold Approximation and Projection [UMAP]), we detected 2 clusters of patients (Figure 6A). Cluster A consisted largely of patients from the lowest-risk lung injury subtype 1, whereas Cluster B consisted largely of patients from the sicker lung injury Subtypes 2, 3, and 4 (Figure 6B). Patients in Cluster B were twice as likely to have required oxygen immediately prior to BAL (50.3% versus 29.1%; 66 of 131 versus 23 of 79; *P* = 0.003; Figure 6C) and, when analyzing the most recent encounter for each patient, were twice as likely to be dead or require ongoing mechanical ventilation 7 days after BAL (37.0% versus 17.2%; 40 of 108 versus 10 of 58, *P* = 0.008; Figure 6D). Consistent with the biologic themes identified by other methods above, Cluster B differentially expressed 9,784 genes in BAL (|LFC| ≥ 1, FDR-adjusted *P* < 0.05) representing AM depletion, lymphocyte activation, and epithelial injury, as well as 696 genes in blood, representing EMT and endothelial activation (Supplemental Data Files 24 and 25). Thus, this unified multi-omics approach demonstrates that post-HCT lung injury can be conceptualized as 2 large groups, with group B harboring mostly lung injury Subtypes 2, 3, and 4 and showing greater illness severity and worse clinical outcomes.

## Discussion

In this study, we compared 210 BAL and blood transcriptome pairs from pediatric patients with HCT with lung injury. We identified minimal cross-site transcriptome correlation at the level of individual gene expression, gene set enrichment scores, cell type fractions, and T and B cell receptor clonotypes. Instead, we uncovered unique site-specific transcriptome networks, suggesting body site compartmentalization of injury response. BAL signatures of mortality reflected immune perturbations, epithelial injury, and hypoxia signaling. Instead of mirroring the pulmonary compartment, peripheral blood transcriptomes showed broad signatures of endothelial injury, DNA repair, neurohormonal activation, and altered cellular metabolism, thus expanding our understanding of the systemic processes involved in post-HCT lung injury.

Other investigators have identified a lack of BAL-blood correlation for cell fractions and cytokines in patients with COVID-19, asthma, and HIV-related lung disease, although transcriptomic comparisons are sparse (15–21). Data in patients with HCT are rare, although Omdahl et al. (22) showed in a nonhuman primate model of GVHD that peripheral and lung-specific T cells have notably different TCR repertoires and even different site-specific T cell transcriptomes when TCRs are shared. While the lung is well known to have a robust local immune repertoire, during lung injury, there is degradation of the alveolar-capillary membrane with transmigration of bone marrow–derived immune cells, including neutrophils and monocytes, into the alveolar space (23, 24); thus, we were surprised to see the starkly absent cross-site correlation. Since the peripheral blood represents a composite of circulating blood cells as well as signals from all tissues in the body, it is possible that the effect of lung disease on the blood signal was diluted by extrapulmonary processes. It is also possible that poor post-HCT systemic immune function and/or exogeneous immunosuppression limited systemic immune responses to pulmonary processes. Overall, this strongly suggests that transcriptome measurements obtained from peripheral blood cannot be used as a proxy for analogous pulmonary processes, and it challenges the current clinical paradigm of assessing pulmonary inflammation using blood-based biomarkers. Whether more durable markers of pulmonary processes, such as protein biomarkers, can be detected in blood to guide pulmonary diagnosis and treatment remains an ongoing question in the field (25–27). However, the prognostic and predictive utility of such approaches in post-HCT GVHD lend optimism to this search (28–30).

While an overlap between BAL and blood pathways of disease was minimal, we did note a few exceptions. A top candidate gene related to post-HCT lung injury in our data was *CEACAM6*, which was upregulated in both BAL and blood of nonsurvivors and also tightly coregulated with BAL expression of numerous genes related to platelet signaling and endoplasmic reticulum stress. *CEACAM6* is expressed on the cell surface of respiratory epithelium, including airway secretory cells and alveolar epithelial cells (31); is upregulated after numerous forms of lung injury; and serves as an intercellular adhesion, antiapoptotic, and surfactant protective molecule (32–36). Future studies addressing the biological role and biomarker utility of *CEACAM6* are warranted.

In addition, we identified a shared BAL-blood transcriptomic signature in lung injury subtype 3, where both the BAL and blood showed upregulation of pathways related to EMT and collagen biosynthesis. Post-HCT fibrosis is a feature of sclerotic GVHD as well as post-HCT bronchiolitis obliterans syndrome and is notoriously difficult to diagnose in the lungs due to the insidious onset and late imaging findings (37–39). Thus our findings could suggest a pathway where detection in peripheral blood might provide insight into pulmonary processes, which could be leveraged for improved diagnostics. Given the lack of tissue for histologic examination, it remains unclear if these patients truly had pulmonary fibrosis, but this can be explored in the future. In addition, it remains unclear whether the detection of this signal in blood represents a synergized systemic response or is simply “leakage” of the pulmonary signal into the blood compartment. Future work is needed to define and characterize the possibility of pulmonary and extrapulmonary fibrosis in this population.

Given these findings, it is likely that the field of post-HCT lung injury will need not only pulmonary-targeted diagnostics, but pulmonary-targeted therapeutics as well. Especially given the serious off-target effects of many immunomodulatory medicines, inhaled therapies possibly coupled with nanoparticles for durable delivery hold high promise for local effect with the least off-target toxicity (40–42). However, it remains unclear whether targeting the identified blood-specific pathways associated with disease could actually improve pulmonary outcomes. In support of this possibility, it has recently been shown that targeting intestinal health can improve respiratory health, perhaps through the gut-lung axis (43, 44).

In summary, by comparing 210 paired BAL-blood transcriptomes obtained after pediatric HCT, we identified surprisingly little cross-site correlation in gene expression. Instead, we identified unique site-specific signatures of disease, suggesting compartmentalization of injury-response. These findings strongly support the need for pulmonary-based diagnostics and therapeutics and also question the exclusive use of peripheral blood testing to guide clinical care in patients with lung injury post-HCT.

## Methods

### Sex as a biological variable.

Our study included male and female human participants. Since BAL and blood samples were paired, each patient served as their own control. Similar findings are reported for both sexes.

### Patients.

As previously described (7), participating pediatric centers screened all patients with HCT preparing to undergo clinically indicated bronchoscopic BAL for diagnostic assessment of pulmonary disease (NCT02926612).

### Biospecimen collection.

Bronchoscopy and BAL were performed at the discretion of the treating team using local institutional protocols. All BAL samples were obtained by pediatric pulmonologists trained in fiberoptic bronchoscopy with anesthesia provided by anesthesiologists or critical care physicians. Lavage protocol was not dictated by the study but typically involved 3–6 aliquots of 10 mL sterile saline inserted into diseased areas of the lung as determined by preceding chest imaging or physical exam. Percent of lavage returned was not routinely documented, and lavage aliquots were typically pooled by the clinical team immediately after collection. After aliquoting for clinical testing, excess lavage remained unfractionated and was placed immediately on dry ice, stored at –70ºC until processing. Blood was collected during the BAL procedure, typically within 30 minutes of the lavage; 2.5mL whole blood was collected directly into a PAXgene tube, which was inverted 5–10 times and stored per the manufacturer’s instructions at –70°C until processing.

### Clinical protocols and data collection.

Clinical microbiologic testing was determined by the treating team and typically included culture for bacteria, fungus, and acid-fast bacteria; multiplex PCR for respiratory viruses; galactomannan antigen; and cytology for *Pneumocystis jirovecii*. Additional molecular diagnostics such as PCR for atypical bacteria or fungi were used at the discretion of the site. After BAL, supportive care protocols were determined by the treating team. Patient demographics, medical history, and transplant-specific data were documented by trained study coordinators at each site. The most recent ANC and ALC measured clinically prior to BAL were documented. Results of clinical microbiologic testing on BAL were documented and not considered complete until 4 weeks after collection. Patients were followed until hospital discharge with no loss to follow-up.

### RNA extraction.

BAL underwent RNA extraction as previously described (7). Briefly, 200 μL of BAL was combined with 200 μL DNA/RNA Shield (Zymo) and 0.5 mm glass bashing beads (Omni) for 5 cycles of 25 seconds bashing at 30 Hz, with 60 seconds of rest on ice between each cycle (TissueLyser II, Qiagen). Subsequently, samples were centrifuged at 16,000*g* for 10 minutes at 4°C, and the supernatant was used for column-based RNA extraction with DNase treatment according to the manufacturer’s recommendations (Zymo ZR-Duet DNA/RNA MicroPrep Kit). RNA extraction of PAXgene tubes was performed for this study. Briefly, 1.7 mL of peripheral blood mixed with PAXgene reagent underwent 10 minutes of centrifugation at 1,200*g*, was washed with PBS, recentrifuged for 10 minutes, and the pellet was combined with 100 μL DNA/RNA Shield, treated with Proteinase K, and underwent a magnetic bead–based extraction with DNase treatment according to the manufacturer’s recommendations (Zymo Quick-RNA Magbead Kit).

### RNA-Seq.

BAL underwent RNA-Seq as previously described using the New England Biolabs Ultra II RNA Library Prep Kit (7). Both BAL and peripheral blood RNA underwent sequencing library preparation using miniaturized protocols adapted from the New England Biolabs Ultra II RNA Library Prep Kit (45). Reagents were dispensed using the Echo 525 (Labcyte) and underwent Ampure-XP bead cleaning on a Beckman Coulter Biomek NX^P^ instrument. The peripheral blood libraries received treatment with rRNA and globin-depleting FastSelect reagent. Libraries underwent 19 cycles of PCR amplification and size selection to a target 300–700 nucleotides (nt), and they were pooled to facilitate approximately even depth of sequencing. Twenty-five picograms (pg) of External RNA Controls Consortium (ERCC) pooled standards were spiked in to each sample after RNA extraction and before library preparation to serve as internal positive controls (Thermo Fisher Scientific, 4456740). In addition, to identify contamination in laboratory reagents and the laboratory environment, each batch contained 2 samples of 200 μL sterile water and 6–8 samples of 200 μL HeLa cells taken from a laboratory stock and processed identically to patient samples, in order to account for laboratory- and reagent-introduced contamination. These samples were processed at the same time as the patient BAL samples in order to use the same lot of reagents and minimize batch effect on control samples. Samples were first sequenced at shallow depth on an Illumina iSeq instrument; *n* = 4 samples were removed due to low sequencing quality, and the remaining samples were pooled across lanes of an Illumina NovaSeq 6000 or NovaSeq X instrument and sequenced to a target depth of 40 million read-pairs with sequencing read length of 125 nt. Resultant fastq files underwent alignment to hg38 (*STAR*), with mitochondrial, ribosomal, and non-protein-coding transcripts excluded, leading to detection of a median 18,341 protein-coding transcripts per BAL (IQR, 17,052–18,755) and a median 14,146 transcripts per blood sample (IQR, 13,339–14,560). The dataset was then further subset for samples with > 50,000 total reads to protein-coding genes and genes present in > 25% of samples (9 blood samples were removed).

### Statistics.

Genes differentially expressed by body site were identified by fitting negative binomial generalized linear models to body site with patient grouping as a random effect (edgeR) (46). Genes differentially coexpressed by body site were analyzed at the pathway level by first creating gene set enrichment scores to the MSigDB Hallmark pathways (gsva) (47), calculating gene set–gene set correlation within each site, and then contrasting the correlations across BAL and blood (DGCA) (48). Gene expression correlation across body sites was assessed by subjecting gene counts to variance stabilizing transformation (DESeq2, vst) (49) and then calculating genewise Spearman correlations using paired values. This analysis was repeated using gene set enrichment scores (gsva). We then used CIBERSORTx (50) to impute cell type fractions in BAL using the Travaglini et al. lung cell atlas (51) and in blood using the Schulte-Schrepping et al. blood cell atlas (52). We then tested for correlation between BAL and blood cell fractions using Spearman correlations. ImReP was used to identify T and B cell receptor repertoires (53). All analyses involving ≥10 comparisons were subjected to FDR adjustment to address multiple-hypothesis testing, and statistical significance was assessed at the level of FDR-adjusted *P* < 0.05.

Contrasts were then repeated by survival status (using each patient’s most recent encounter) and by lung injury subtype. Here, site-specific gene-to-gene correlation networks were created by identifying genes correlated in 1 body site (Spearman rho ≥ 0.5) and not correlated in the other body site (Spearman rho < 0.1) with a significant contrast of FDR-adjusted P < 0.05 (DGCA). Gene networks were plotted using Cytoscape v3.10.3 (54). Genes involved in each gene network were then ranked by Hub Score (igraph) (55) to identify most central genes, and were summarized at the pathway level using gene set enrichment (ClusterProfiler) (56). We integrated BAL and blood transcriptomes by applying variance-stabilizing transformation (vst, DESeq2) (49) to BAL and blood transcriptomes followed by MOFA (57), dimensionality reduction (UMAP), and k-means clustering (cluster). Data were visualized using volcano plots (EnhancedVolcano), box-and-whisker violin plots (ggplot), heatmaps (pheatmap), and gene set enrichment plots (ClusterProfiler).

### Study approval.

Patients or their guardians were approached prospectively for consent under local IRB approval at each site (UCSF IRB nos. 14-13546 and 16-18908) in accordance with the Declaration of Helsinki, and permission was obtained to collect leftover BAL fluid as well as paired blood.

### Data availability.

Raw sequencing files and instructions to request download are available under controlled access on NIH dbGaP (https://www.ncbi.nlm.nih.gov/projects/gap/cgi-bin/study.cgi?study_id=phs001684.v3.p1). Individual-level data are available indefinitely. Code and processed anonymized individual-level data files are available on GitHub (https://github.com/zinterm/pedBMT_BALseq; commit ID 4beb6dd). Values for all data points in graphs are reported in the Supporting Data Values file.

## Author contributions

Conceptualization: MSZ, CCD, EMP, and JLD. Methodology: MSZ, CCD, EMP, and JLD. Investigation: EMP, EE, MYM, GR, MRS, JB, HK, JM, PJS, CMR, JJA, PLM, CH, EMK, MQ, HAA, AKK, GDEC, JRH, JSK, KG, RH, CND, TCQ, PC, NRL, JCF, KMM, PS, TBM, BH, AAM, DBD, MPH, GAY, MAP, CCD, JLD, MSZ. Visualization: MSZ, EMP, and JLD. Funding acquisition: MSZ, JLD, MAP, and CCD. Project administration: MSZ, JLD, and CCD. Supervision: MSZ and JLD. Writing – original draft: MSZ, CCD, EMP, and JLD. Writing – review & editing: EMP, EE, MYM, GR, MRS, JB, HK, JM, PJS, CMR, JJA, PLM, CH, EMK, MQ, HAA, AKK, GDEC, JRH, JSK, KG, RH, CND, TCQ, PC, NRL, JCF, KMM, PS, TBM, BH, AAM, DBD, MPH, GAY, MAP, CCD, JLD, MSZ

## Supplementary Material

ICMJE disclosure forms

Supplemental data sets 1-11

Supplemental data sets 12-25

Supporting data values

## Figures and Tables

**Table 1:**
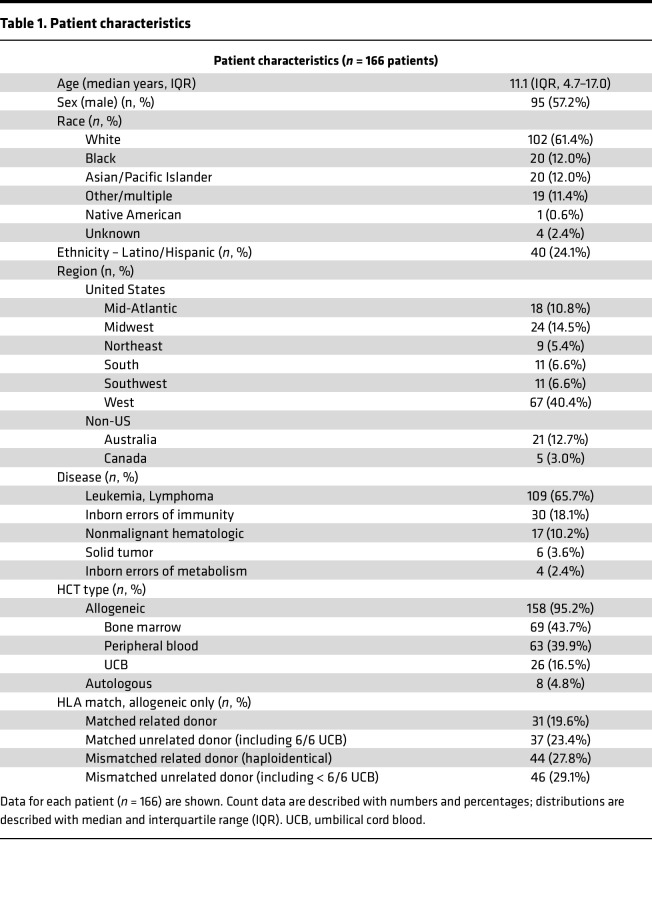
Patient characteristics

**Table 2:**
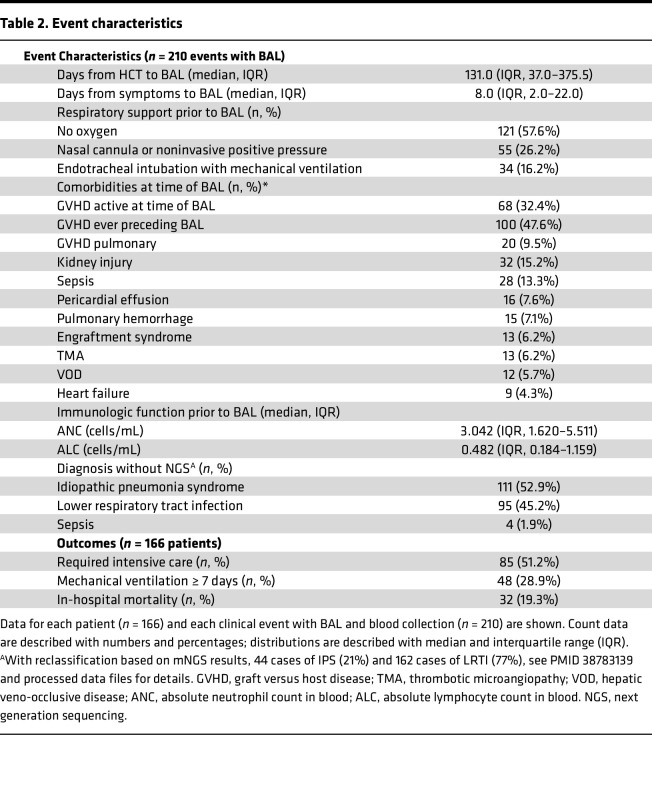
Event characteristics

**Table 3:**
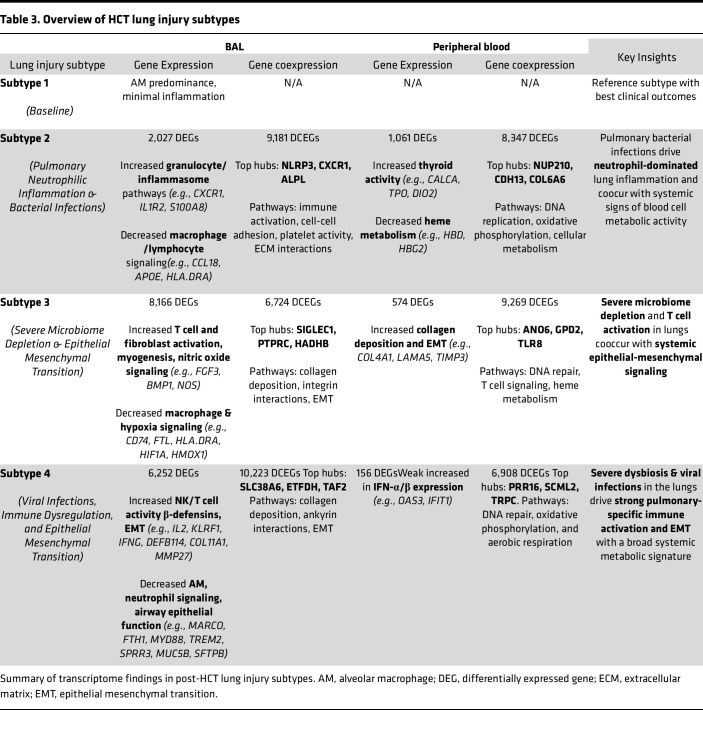
Overview of HCT lung injury subtypes

## Version 1 07/22/2025

In-Press Preview

## Version 2 09/09/2025

Electronic publication
